# Reshaping the equitable and inclusive access to healthcare: A qualitative study

**DOI:** 10.1016/j.cegh.2024.101544

**Published:** 2024

**Authors:** Jignesh Patel, Sangita More, Pravin Sohani, Shrinath Bedarkar, Kamala Kannan Dinesh, Deepika Sharma, Sanjay Dhir, Sushil Sushil, Raj Shankar Ghosh

**Affiliations:** a Jivika Healthcare, Maharashtra, India; b Department of Management Studies, Indian Institute of Technology, Delhi, India; c Public Health Consultant, New Delhi, India; d Jindal Global Business School, OP Jindal Global University, Sonipat, Haryana, India

**Keywords:** Health care, Equity, MMUs, Qualitative, Sustainability, Inclusion

## Abstract

**Background:**

Improving equitable access to healthcare requires innovative interventions and strengthening a service innovation operational model to achieve transformative change and bring sustainability to public health interventions. The current study aims to identify the components of the Mobile Medical Units (MMUs) operational model as an innovative intervention to provide equitable and inclusive access to healthcare.

**Methods:**

The study used qualitative research to identify the components of the operational model of MMUs for primary healthcare in future. Data has been collected via semi-structured in-depth interviews with 103 healthcare professionals from six states representing India's Tier I, Tier II, and Tier III regions. A thematic analysis was performed to examine emergent salient themes.

**Results:**

The study identified and examined scalability, affordability, replicability, and sustainability as the four critical components of the operational model of MMUs. The findings of the study indicated that MMUs with these four components played a vital role in COVID-19 immunization, especially in resource-limited settings. The study found that MMUs are a cost-effective and scalable healthcare delivery model that can be easily replicated in primary healthcare service delivery.

**Conclusion:**

The findings underscore the significant role of MMUs in addressing healthcare disparities, particularly in resource-limited settings. The adaptability and cost-effectiveness of MMUs make them an ideal solution for primary healthcare delivery, especially in Tier I, II, and III regions of India. It lays a foundation for future research and policy-making, emphasizing the need for innovative, equitable, and sustainable healthcare delivery models like MMUs to transform and strengthen healthcare systems globally.

## 1 Introduction

Equitable access to healthcare for rural, tribal, and underprivileged people has been an emerging area of interest for researchers, academicians, and policymakers worldwide.^1^ Healthcare practitioners have been striving to adopt cutting-edge strategies to suit the changing requirements of our population's health equitably and sustainably.^2^ The limited and inconvenient access to healthcare facilities by immunocompromised, hesitant people and underserved communities leads to reduced vaccine uptake, the low effectiveness of immunization programs, and the failure of other healthcare programs.^3^^,^^4^ In this context, Mobile Medical Units (MMUs) hold a significant promise to provide equitable and convenient access to various healthcare facilities, including vaccinations, to the underserved and immunocompromised population.^5^ MMUs may be the only way for some individuals to access critical medical care.^6^ MMUs are an innovative model of health services delivery that play a crucial role in providing healthcare to vulnerable populations, especially in areas with limited resources.^7^^,^^8^ Many people may not have access to primary health care and immunization programs without mobile clinic services.

MMUs are an underutilized resource within the healthcare system.^9^ The mobility and versatility of MMUs make them excellent partners for combating pandemics like COVID-19.^10^ In many regions of the world, MMU programs already play significant, though undervalued, roles in the healthcare system.^11^ They fill gaps in the healthcare safety net, reaching socio-economically underserved populations in urban and rural areas, and offer versatility in setting a damaged or inadequate healthcare infrastructure. They also provide access to healthcare, especially for displaced or isolated individuals. MMUs have been struggling to gain widespread support in developing countries, despite mounting evidence of their distinctive usefulness and ability to be highly adaptable as a treatment model. This has led to a squandered opportunity to use these previously established and reliable initiatives to remove access hurdles faced by under-resourced areas and to deploy MMU during national emergencies like the COVID-19 epidemic.

MMUs are a well-established type of community-based service delivery that bridges the gaps in healthcare safety nets and reaches communities that are socially and economically marginalized in urban and rural locations.^12^ MMUs are a crucial tool for providing high-quality care to medically disadvantaged communities in various regions of the world. With a cutting-edge approach to healthcare delivery, MMUs can potentially reduce health inequities among underprivileged communities and those with chronic illnesses. Several studies have found that MMUs have a more significant impact when they provide emergency treatment, do preventative health screenings, and start managing chronic diseases.^13^^,^^14^ MMUs can provide personalized, highly effective, reasonably priced health care that flexibly adapts to the community's changing requirements by entering communities directly and utilizing existing community resources.

In addition to the requirement, the highlighted gaps in the literature provide adequate grounds for conducting an empirical study on enhancing the scalability and sustainability of mobile health clinics. Extant literature showcases that the primary reasons MMUs encounter challenges are the lack of a clearly defined framework for these clinics in healthcare providers’ plans and present policymaking.^7^^,^^8^^,^^15^ The main objective of this study is to examine the critical factors necessary to construct an operational model for mobile health clinics that may be used for other public health initiatives in the future.

The study has incorporated a three-pronged strategy in this remark to identify and examine various ways towards the sustainability and replicability of MMUs in the healthcare system: The first stage identifies the components of MMUs' operational model, which can be replicable for primary healthcare in the future. An extensive literature review has been conducted to identify the components of MMUs' operational model. In the second stage, the elements identified have been examined to determine their impact on immunization performance through semi-structured in-depth interviews. Thus, in this direction, Jivika Healthcare's service innovation support, VaccineOnWheels (VOW), is considered among several organizations catering to vaccination services through MMUs. The working model exhibited by VOW is exemplary, as the MMUs under the organization are equipped with all the necessary supplies and equipment required for immunization, and the health workers are trained to provide immunization services safely and effectively. Under the third stage, the study delineates strategic recommendations for strategic professionals to strengthen and sustain MMUs in primary healthcare in the future.

## 2 Methods

### 2.1 Case organization

In the context of MMUs in India, the study has considered VaccineOnWheels (VOW) as the case organization. In 2019, Jivika Healthcare Private Limited launched Vaccine on Wheels-First India's Doctor-based Mobile Vaccination Clinic to “ensure access to quality vaccination for all” and aimed to reduce inequality and increase immunization reach, provide low-cost vaccines, and create awareness of immunization.

This program helped to understand the gaps in the vaccine delivery model from close quarters and identify various issues faced by diverse stakeholders, primarily infants/caregivers/parents, in getting vaccinated. VOW has made vaccines accessible to the elderly, individuals with disabilities, female sex workers, tribal communities, rural communities, street vendors, maids, slum residents, frontline workers, bedridden, and school children, among other vulnerable segments of society. They have also provided door-to-door service to those who could not reach the vaccination center. They have served the people residing in remote locations of the six states through more than 200 mobile vaccination units. Under the unique framework of the Public-Private Partnership Model (PPP), vaccination is administered at a reduced cost for beneficiaries with vaccines provided by the government. The PPP model enables stakeholder collaboration across industries under Corporate Social Responsibility (CSR), government, and Non-Governmental Organizations (NGOs) to share a commitment to making vaccination services available even at grassroot levels. This initiative shall help India achieve higher immunization penetration by getting faster acceptance towards vaccination, giving more convenience, and reducing the cost of seeking service with zero travel cost, travel time, and low wages.

### 2.2 Methodology

The extant literature underscores the significance of qualitative research in public health, and this study adopted an exploratory qualitative research design to examine the concepts and processes related to MMUs.^16^^,^^17^ This design is particularly apt for understanding the scalability, affordability, sustainability, and replicability of such clinics in enhancing immunization coverage in selected states and districts.

The methodology was enriched by incorporating a systematic content analysis of the interview data.^18^ The verbal responses were transcribed verbatim after conducting group and in-depth interviews with key stakeholders of the VaccineOnWheels (VOW) initiative. This transcription served as the basis for an in-depth content analysis. Initially, a familiarization phase involved thoroughly reading the transcripts to identify preliminary themes related to the research questions. Subsequently, a coding process was implemented, tagging text segments with specific codes that encapsulated critical concepts related to the study's focus areas.

This coding led to identifying recurring themes, which were then reviewed and refined for coherence and relevance to the research questions. The themes were defined and named, ensuring they accurately captured the essence of the stakeholders' perspectives on the scalability, affordability, sustainability, and replicability of mobile clinics.

### 2.3 Data collection

In-depth, semi-structured interviews were conducted across selected states and districts, targeting a diverse group of healthcare stakeholders. These stakeholders included health officers, grassroots workers, mobile clinic operators, NGOs, strategic partners, policymakers, and other support staff. The interviews delved into various aspects of the immunization program, focusing on scalability, affordability, sustainability, and replicability. Additionally, stakeholders shared their experiences and insights on challenges, innovations, and best practices contributing to high immunization rates.

A total of 102 respondents, directly involved in the mobile clinic vaccination campaign, provided valuable data. These respondents represented a cross-section of roles within the healthcare system and hailed from states where VOW was operational, including Jharkhand, Maharashtra, Meghalaya, Karnataka, Telangana, and Tamil Nadu.

With consent obtained for recording and transcribing the interviews, the data was methodically processed for content analysis. The transcribed data was meticulously coded to identify patterns and themes. This coding was not merely descriptive but also interpretive, aiming to extract deeper meanings and insights from the stakeholders' narratives. The analytical process was iterative, with ongoing refinement of themes to ensure they precisely reflected the complexities and nuances of the stakeholders' experiences and perspectives. This comprehensive analysis was critical for developing a nuanced understanding of the factors affecting the success of mobile-based vaccination clinics in the selected regions.

The distribution of cities across different tiers in the states of Maharashtra, Karnataka, Tamil Nadu, Jharkhand, Meghalaya, and Telangana, are categorized based on their urban tier classification. In Maharashtra, the cities are variedly placed with Mumbai and Pune in Tier 1, Aurangabad and Nashik in Tier 2, and Nanded, Latur, and Thane in Tier 3. Karnataka is represented by Bengaluru, a Tier 1 city. Tamil Nadu has Tirunelveli in Tier 2, while Ranipet and Tirupathar are in Tier 3. Jharkhand's representation includes Chatra, West Singhbhum, and Lohardagga, all classified as Tier 3 cities. Meghalaya is represented by East Khasi Hills, also in Tier 3. Lastly, in Telangana, both Sangareddy and Wanaparthy are categorized as Tier 3 cities. This classification provides a clear understanding of the urban hierarchy and demographic distribution across these states, which is crucial for contextualizing various developmental and policy-related initiatives.

## 3 Results

### 3.1 Respondents’ profile

**Fig. 1:**
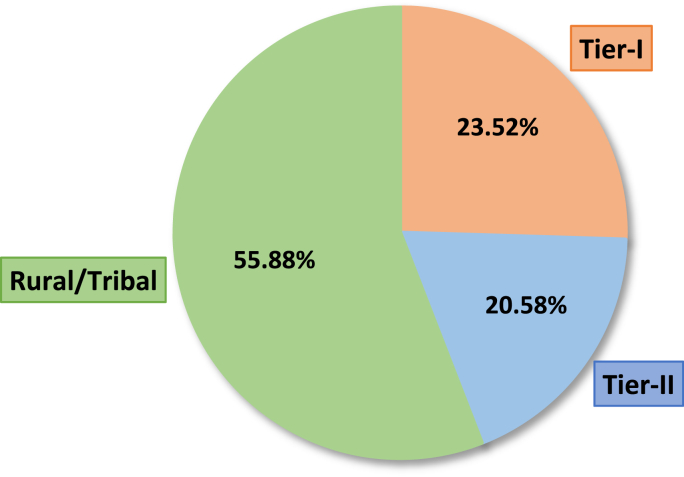
Composition of interviews from different regions.

A total of 103 interviews were conducted in six selected states of India. Participants included Accredited Social Health Activist (ASHA) employees, Gram Sevaks, District Health Officers (DHOs), Taluka Health Officers (THOs), Chief Health Officers (CHOs), Senior Medical Officers (SMOs), Assistant Health Officers (AHOs), Medical officers (MOs), Campaign coordinators, and other personnel involved in this immunization program. More than 50% of the participants were medical officials. From the total number of interviews, around 56% of the respondents were working in rural or tribal areas, 21% were working in Tier-II districts or cities, and the remaining 23% represented the health workers from Tier-I cities or districts. Fig. 1 shows the composition of interviews conducted from different regions.

### 3.2 Findings from qualitative analysis

Fifteen of the twenty-four participants from Tier-I cities and districts are from Maharashtra, while nine are from Bengaluru, Karnataka. The responses from Tier-I areas comprise several medical officials and other essential support personnel who supported the vaccination program. Before analyzing the interviews, there was a preconceived belief that Tier-I healthcare systems are superior to Tier-II and rural healthcare systems. Even if the healthcare system is improving, gaps may be addressed to enhance the system further. One of the objectives of this qualitative analysis is to comprehend the operation of the mobile vaccination program in Tier-I locations in collaboration with government agencies and other stakeholders. The analysis would highlight the principal role of the many stakeholders, the significant impacts noticed, the essential operation comprising planning and execution, the problems encountered, and concluding remarks on the program's viability and scalability. Tier-II regions account for twenty-one percent of overall participants. Similar to the composition of respondents in Tier-I regions, more than fifty percent of respondents in this domain are medical officials. The remainder of the participants reflects those participating in the coordination and assistance of the immunization campaign. Fifty-seven of the 103 interviewees are from rural or tribal locations. Approximately 56 percent of the study's participants are from rural and tribal communities. Since the objective of the mobile vaccination team was to vaccinate as many people as possible in rural and tribal communities, the team was effective in this region.

The interviews were observed and evaluated following the status of the regions. Select excerpts from the interviews have been displayed in Table 1. The sub-dimensions of significant factors are identified and cross-compared with all the districts representing different regions. The detailed cross-comparison of each dimension is given in Table 2. It also showcases different stakeholders’ perceptions and consolidated responses. Following cross-comparisons of dimension, significant problems faced by the mobile clinic team are classified and segregated according to a different region. Table 3 displays critical problems and major interventions taken by the VOW team to resolve those issues.

**Table 1:** Select excerpts from interviews.

Components and features	Tier-I	Tier-II	Rural/Tribal
**Roles and responsibilities**	• Local public health governance • Ward (vaccination) monitoring • Handling vaccination centers • Appointing staff and data entry operators for the vaccination program	• Identifying the areas with less coverage • Scheduling the sessions • Arrangements at the vaccination center • Deploying staff at the center to assist in the vaccination program	• Identifying the area with limited coverage and scheduling even with just 50 people to be vaccinated • Awareness to be created to mobilize the rural and tribal population • Solving their doubts related to post-vaccination side effects, if any • Taking the help of the local leaders and Sarpanch to mobilize the population
**Major impact**	• Adequate immunization in slum areas • Good outreach to beneficiaries • Resolved the issue of transport and logistics • Trained workforce and door-step service approach • Significant increase in the vaccination rate	• Marginal groups such as sex workers and trans genders were vaccinated • Good outreach to beneficiaries such as street vendors, old age people, bedridden patients • The vaccination drive was conducted at the convenience of beneficiaries- early morning, late evening • Vaccination coverage was significantly increased • School-going children above the age of 12 are getting vaccinated	• Significant rural and tribal population is covered with the help of MMUs • Vaccine at their door-step was possible because of the MMUs • Most people are daily wage earners, so for them, MMUs were available even in the early morning and at night • A visible increase in vaccine coverage was observed, with more than 90%
**Coordination, planning, and execution**	• Microplanning for the program before 15 days or one week • Communication, either through phone calls or instant messaging groups • Identified and selected areas for vaccination • Regular reporting of vaccination status • Frequent data monitoring (vaccine availability and list of beneficiaries) and revision of the program plan	• Scheduling was done in advance by the health officers for the unvaccinated blocks. • Awareness regarding the vaccination drive was announced in the area a day before • Required vaccines were shared with the MMUs • Sessions were created, and the MMUs	• entered data • The health officers did scheduling for the unvaccinated blocks Plan was made in advance and shared with the mobile clinic team. • Asha worker assisted the MMUs in the vaccination drive • Reporting was done on the sessions created by the data operator of the mobile clinic's team • Physical data were also maintained simultaneously
**Challenges**	• Hesitancy exhibited by few communities in the slum areas • Initially, very few beneficiaries showed a willingness to vaccination • Difficulty in mobilizing the beneficiaries • Substantial effort in implementing appropriate IEC strategies • Technical issues such as internet connectivity and poor communication • The COWIN portal was down on some occasions • Few beneficiaries were skeptical about vaccines and their adverse effects	• Mobilizing beneficiaries was challenging • Due to the working hours, there were sometimes fewer beneficiaries available for vaccination . • A significant concern of the people was related to the vaccine's side effects. • The problem related to the network issue was another concern because of which it was not easy to enter data immediately on the COWIN portal .	• A significant challenge was checking the beneficiaries' availability as they were daily wage workers and were mostly unavailable during the daytime. • People had many hesitations and were unwilling to take the vaccine • It was not easy to reach the interiors of the remote villages • The tribal and rural population was scanty and scattered, which required additional planning to schedule the vaccination sessions • Because of less number of people available for vaccination at times, vials also remained unused
**Primary healthcare feasibility**	• Initialized discussion for primary healthcare concerning the age group 0–5 years • Should collaborate with municipality and local organizations to work for routine immunization • The support from mobile teams will be an added advantage.	• Primary healthcare can be conducted in collaboration with MMUs for better reach. • MMUs will provide an added workforce to cover a vast population. • MMUs' professional teams can aid in supporting the primary healthcare initiative.	• Association with MMUs for primary healthcare can be highly beneficial for rural and interior tribal areas. • Since hard-to-reach areas have limited transportation, MMUs can aid in providing door-to-door vaccination coverage to children below the age of 5
**Program expansion**	• Support from the mobile vaccination team can enhance the vaccination rate and coverage. • Many healthcare centers in India lack logistics support, and the VOW team can support these cases. • It will be helpful to vaccinate areas that are hard to reach	• MMUs can help in the coverage of the school-going population above the age of 12 • The efficiency of MMUs can be utilized for giving booster doses to the beneficiaries • Reaching the unreachable can be achieved through their help	• MMUs can provide the required logistics to serve rural areas, such as providing ambulance • More MMUs can serve the underserved and can help in reaching to the last mile • Efficient utilization of MMUs can increase the vaccination coverage

**Table 2:** Cross-comparison of significant factors of immunization (stakeholder-wise).

*Stakeholders →*	Grass rootworkers (Asha, VHN, ANM, etc.)	Health Officers (CHO, MO, DHO, etc.)	Admins (DDM, Establishment Officer, State Coordinator, etc.)	Partners and Community Leaders (NGO, Sarpanch, Gram Sevak, etc.)
Factor ↓				
**Top-level management**	The administrative support was satisfactorily provided, including timely assistance and guidance. There was legal and technical assistance and training, including guidelines to access and manage data in the CoWIN portal.	Health officers were prompt enough to monitor, supervise and evaluate the program. They also identified and selected the regions for the vaccination program. They also ensured a regular supply of the required vaccines and other medical equipment.	Administrative support was adequately available to facilitate the vaccination program. The required clearances were fast-tracked, and necessary approvals were timely made. All the procurements were done under NHM (National Health Mission).	NGOs and community leaders actively mobilized the beneficiaries for participation in the immunization program. They had a more significant impact on the local population.
**Coordination, Planning, & Execution**	Their main task was to identify regions and sites for conducting vaccination programs. The grass root workers also prepared the list of beneficiaries. The other essential task was to enter the data and make registrations. Daily reporting of the number of vaccinated people was another important objective.	A typical communication platform (WhatsApp and Phone calls) seamlessly transferred knowledge. Teams were formed, keeping the strength of the beneficiaries in mind. The sessions were planned and scheduled according to the availability of the beneficiaries. Session planning and scheduling There were regular reviews of the status of vaccination campaigns. A review of vaccination coverage was also conducted simultaneously.	The initiation of the campaign in select regions based on vaccination coverage was well-planned and coordinated. Assigning of the blocks and respective health officers for vaccination through MMUs was also conducted. Facilitated the immunization program diligently.	NGOs like ‘Give India’ ensured there was enough funding for the execution of the vaccination program effectively. NGOs supported the mobilization of the beneficiaries. There was the active involvement of the NGOs in community engagement. They helped in motivating the local population to get themselves vaccinated.
**Challenges**	The major challenge revolved around vaccine hesitancy. Immobilization was highly prevalent. Health illiteracy among the local population was also one of the critical reasons for the lower vaccination rate initially. Rumors regarding the ill effects of the vaccination were considered one of the significant reasons. Since the transportation service in the local areas could be more robust, leading to a lower vaccination rate.	There were issues related to the infrastructure. One of the major issues revolved around the technical glitch. At times there was a lack of assessing the vaccination program. Sometimes it was observed that there needed to be more appropriate preparation for IEC.	Specific infrastructural issues acted as a significant challenge during the vaccination program. However, most challenges were handled diligently, and due arrangements were made to combat the infrastructural challenge. For example- the supply chain and logistics were well coordinated.	The crucial challenges revolved around the immobilization of the beneficiaries. There were substantial efforts to eradicate the rumors about the side effects of vaccination. Especially in rural areas, such rumors were widespread because the decision to get vaccinated was delayed.
**Impact**	The grass root workers significantly impacted the reachability of the vaccination program. Since they knew the local demographics, it helped in reaching the beneficiaries.	The health officers significantly impact greater reachability, a higher vaccine uptake, and broader immunization coverage.	It immensely impacted reachability, and they ensured that the unreachable was reached. The immunization coverage was increased, leading to a successful vaccination program.	The vaccine uptake was increased with the involvement of the community as the local population was more comfortable with them. Overall, community engagement was increased, and a higher number of people were vaccinated.
**Strategic views**	As the vaccination program grows, more support is required in terms of the number of support staff and MMUs, especially for the coverage of school-going children. With the aid of ASHA and ANM workers, vaccination programs can have greater penetration in the interiors.	The program can be replicated in other states with similar actions but requires thorough training before moving to primary healthcare. Primary healthcare requires a higher level of expertise since children are involved.	The model can also be scaled up by engaging MMUs in other states. This program can be replicated according to the demographics of the concerned states. Regarding routine immunization, already a standard system is well in place. Therefore no primary need is felt for another program to be executed.	It will benefit a more extensive set of populations if implemented nationwide. The model has the potential to conduct primary healthcare as well. As it was observed that health workers during the vaccination program were reaching the door-step, if a similar door-to-door primary healthcare is conducted, more children will be vaccinated.

**Table 3:** Critical problems, intervention, and impact.

	Problems and issues	VOW intervention	Impact
**Tier-I**	Hesitancy in slum areas	The VOW team and local municipal leaders resolved any hesitancy related to vaccination.	More people were willing to take the vaccine.
	Difficulty vaccinating specific populations (migrant workers, sex workers, transgender people etc.)	The VOW team reached their door-step to make them comfortable, as they hesitated to go to the vaccination camps	A more significant number of marginalized populations was covered.
	Difficulty in mobilizing beneficiaries	The team conducted awareness programs, and beneficiaries were given counseling.	People were impacted positively, and the number of vaccinated people grew slowly.
	Server issues	During server issues, data regarding vaccination was registered physically	Physically registered data was uploaded by the VOW personnel once the server resumed, thereby no data was missed
	Rumors about vaccine adverse effects	Any doubts about the vaccine's post-effects were communicated to the beneficiaries.	People were more confident regarding the COVID-19 vaccine
**Tier-II**	Mobilization issues	The VOW team and ASHA workers went door to door to mobilize the population	A more significant number of people were vaccinated.
	Scheduling issues (Clash of session timings with working hours of beneficiaries)	The VOW team visited the beneficiaries in the early morning and late evening hours per their availability.	It led to more excellent coverage and an assurance that no one would be left behind.
	Hesitancy caused by false information about vaccines	Proper counseling sessions were taken place to remove any hesitancy	Beneficiaries were more confident, and gradually the rumors were removed
	Network issues (Data entry on Cowin portal)	If the server was down, the data entry operator physically registered the beneficiaries' details.	Physically collected data was eventually uploaded once the server resumed
**Rural/Tribal**	Unavailability of beneficiaries (daily wage workers)	Early morning and late evening sessions were arranged to vaccinate the beneficiaries	This made it convenient for the local people to get vaccinated
	High vaccine reluctancy	Community engagement (Panchayat leaders, religious heads, schoolteachers)	Local leaders had a more significant impact on the local population, and it led to higher coverage
	Low health literacy	Appropriate IEC strategies were employed to educate rural people	People were more aware and educated regarding the vaccination
	Low reachability	Van and other vehicles were used.	Since the rural area terrain is challenging to reach, VOW ensured more excellent coverage by reaching their door-step
	Population is scattered	Gathered the beneficiaries at a commonplace in their region	Small pockets where people were less in number were vaccinated at their convenience
	Transportation issues (Poor roads and infrastructure)	Avoided rainy days to travel; Departed early to reach the vaccination site on time	VOW team was prompt to address such issues and planned accordingly; thus, reachability increased

After examining the immunization program in Tier-I regions, it was determined that it was well-planned and carried out. The assignments were allotted appropriately, and the project was completed according to the plan-generated schedule. Observing the key obstacles faced by the stakeholders, it was noticed that no significant obstacles were encountered during the program. It indicates that the program ran smoothly in Tier I locations. In addition, it was underlined that primary healthcare programs require minimum support from the mobile vaccination team.

The immunization program in Tier-II regions appeared comparable to that in Tier-I locations. However, there were a few operational differences. In Tier-II regions, it was noticed that the team's contribution was substantial. Recall that the team was also engaged in data management tasks. Concerning obstacles, the team needed more in terms of people management, reluctance, and shortage of workers. In these regions, the expansion and extension of the program make sense. Consequently, Tier-II locations are believed to require a mobile vaccination team for immunization programs.

Finally, the program in rural and tribal regions is analyzed. The program's objectives aligned nicely with tribal and rural regions. Despite vaccination operations progressing, the program encountered reluctance, technical issues, geographical obstacles, and infrastructural difficulties. It has been noticed that it is difficult to vaccinate rural populations using the conventional method. This door-step approach, with the assistance of VOW, was successful. As an extension of this approach, a similar model would be effective for primary healthcare in rural and tribal areas.

Table 1 in the study presents a detailed overview of the roles, impacts, coordination strategies, challenges, and future prospects of the MMUs in the COVID-19 vaccination program across different geographical tiers in India. In Tier-I areas, local public health governance, effective ward monitoring, and a door-step service approach were key in addressing hesitancy and logistical challenges, leading to improved immunization rates in slum areas. In Tier-II regions, MMUs focused on identifying less-covered areas, scheduling convenient vaccination sessions, and ensuring outreach to marginalized groups like sex workers and transgender individuals, resulting in a significant increase in vaccination coverage. In rural and tribal areas, the challenges were more pronounced due to scattered populations and low health literacy. MMUs addressed these through strategies like early morning and late evening sessions, mobilizing the population with the help of local leaders, and deploying vans to reach difficult terrains, substantially increasing vaccine coverage.

Coordination efforts involved microplanning, using communication platforms for information dissemination, and scheduling vaccination drives in advance. Challenges varied across regions but commonly included hesitancy, technical issues, and logistical difficulties. The potential for expanding primary healthcare through MMUs was also recognized, with suggestions for collaboration with local organizations and utilizing MMUs for routine immunization, especially in hard-to-reach areas and for vulnerable populations. The expansion of the program was envisioned to enhance vaccination rates, cover school-going children, and provide logistics support in rural areas. Overall, MMUs have demonstrated their versatility and effectiveness in diverse settings, suggesting their pivotal role in future healthcare initiatives.

Table 2 of the study offers a comprehensive cross-comparison of significant factors influencing immunization, as perceived by different stakeholders, including grassroots workers, health officers, administrators, and community leaders. Grassroots workers, like Asha, VHN, and ANM, were crucial in providing administrative support, including managing data in the CoWIN portal, which was instrumental in overcoming vaccine hesitancy and health illiteracy. Health officers played a vital role in the program's monitoring, supervision, and logistical management, ensuring the regular supply of vaccines and equipment. Administrators facilitated the vaccination program by ensuring timely clearances and handling procurements under the National Health Mission. Community leaders and NGOs, like 'Give India,' were key in mobilizing beneficiaries and increasing vaccine uptake through active community engagement.

Coordination, planning, and execution across all tiers involved tasks like identifying vaccination sites, managing beneficiary lists, session scheduling, and overcoming infrastructural and technical challenges. The impact of these collective efforts was significant, leading to increased reachability and vaccination coverage, especially among marginalized groups and in remote areas. The strategic views of these stakeholders highlighted the need for more support staff and MMUs, especially for school-going children, and suggested the potential for replicating this model in other states for primary healthcare. This comprehensive stakeholder analysis underscores the multifaceted approach required to effectively implement vaccination programs and the potential scalability of such models for broader healthcare initiatives.

Table 3 in the paper delineates the critical problems, interventions by VOW, and their impacts across different tiers, revealing a nuanced approach to addressing diverse challenges in vaccination campaigns. In Tier-I regions, VOW effectively mitigated hesitancy in slum areas and among specific populations like migrant workers, sex workers, and transgender individuals, through tailored approaches like doorstep vaccination and collaboration with local leaders, resulting in increased willingness to vaccinate. They combated server issues by physically registering data and resolved misinformation about vaccine side-effects through clear communication, thereby boosting confidence in the vaccine. In Tier-II areas, door-to-door mobilization by VOW and ASHA workers, scheduling adjustments, and counseling sessions to counter hesitancy and misinformation led to greater vaccine coverage and beneficiary confidence. Rural and tribal regions presented unique challenges, including unavailability of daily wage workers, high vaccine reluctancy, low health literacy, and logistical issues due to scattered populations and challenging terrain. VOW addressed these through strategies like timing vaccination sessions to suit local schedules, engaging community leaders for better outreach, employing appropriate Information, Education, and Communication (IEC) strategies, using vans for increased reachability, and organizing vaccination at convenient locations for scattered populations. These adaptive and community-focused strategies significantly enhanced the reach and impact of the vaccination program, demonstrating the effectiveness of VOW's approach in diverse settings.

## 4 Discussion

Numerous global immunization programs have been implemented to eliminate various diseases. However, the COVID-19 vaccination campaign adopted a different strategy, aiming to vaccinate a large population in a short time to achieve herd immunity. This posed challenges in countries like India, where a significant portion of the population resides in rural and tribal areas, requiring targeted interventions. One such intervention was the implementation of mobile vaccination units, specifically the VOW model, which successfully increased vaccination reachability and facilitated rapid immunization against COVID-19 in rural areas. The scalability, affordability, replicability, and sustainability of the mobile vaccination program were found to be crucial factors affecting vaccination rates and coverage.

**Fig. 2:**
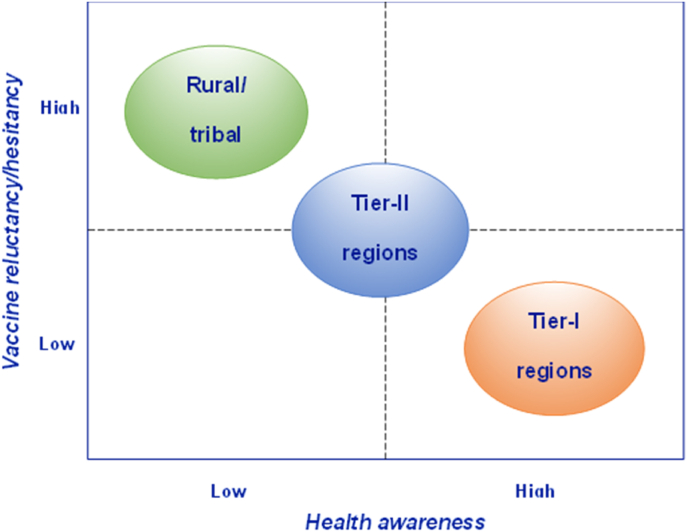
Position of regions in vaccine hesitancy-health awareness matrix.

The study examined the operational aspects of the VOW program in different regions, including Tier-I, Tier-II, and rural or tribal areas, and found that the initiative was successful in all regions, although operational actions varied. Vaccine hesitancy and acceptance which is a critical issue in immunization, was found to be lower in Tier-I regions due to higher health awareness, while rural and tribal regions exhibited higher reluctance towards vaccines and vaccination programs due to prevalent myths and lower health awareness.^19^ Apart from this, multiple perceptions are attached to COVID-19 immunization.^20^ Fig. 2 presents a graphical representation of the regions based on vaccine hesitancy and health awareness.

**Fig. 3:**
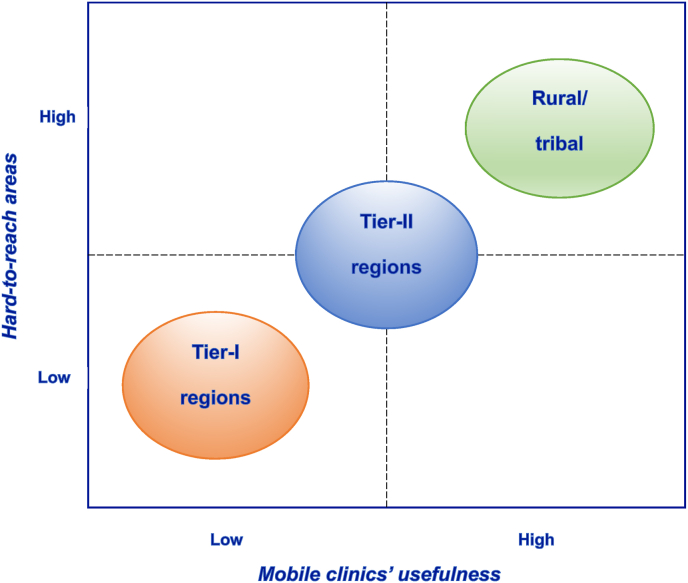
Position of regions in the matrix of hard-to-reach areas vs. MMUs' usefulness.

Furthermore, the study identified that Tier-I regions already had well-performing primary healthcare programs and better infrastructure, resulting in lower utilization of MMUs. However, MMUs effectively delivered routine immunizations in Tier-II regions and addressed the needs of hard-to-reach areas, particularly in rural and tribal communities. Fig. 3 depicts the positioning of regions based on hard-to-reach areas and the usefulness of MMUs.

The qualitative investigation revealed that the mobile clinic approach employed by MMUs has the potential for widespread deployment and expansion beyond COVID-19 immunization. The findings suggest that this innovative approach can be applied to various routine and other healthcare services, necessitating administrative support for national integration. With the support of community healthcare workers and frontline forces, MMUs have the capacity to provide vaccination services to vulnerable populations in both rural and urban areas, benefiting low-income minority communities with significant health inequalities.^21^^,^^22^ Moreover, MMUs can aid in the early-stage detection and prevention of chronic diseases, diagnostic operations, and other healthcare support services.

The mobile vaccination approach, specifically the VOW model, has proven effective as per the findings of the study in reaching underserved populations and facilitating rapid immunization against COVID-19. This innovative strategy has broader implications for routine healthcare services and can be applied on a larger scale to build sustainable mobile clinic programs, benefiting vulnerable communities and improving overall healthcare delivery.

### 4.1 Strategic recommendations

The healthcare industry is currently encouraged to evolve beyond merely providing care access, focusing on value creation through innovative business models. This shift is exemplified by the "M-CLINIC 2.0" framework, which advocates for a holistic and proactive approach to patient care, aimed at achieving optimal health outcomes. This model will leverage existing healthcare resources effectively to fulfill the anticipated health outcomes, marking a significant shift towards a more patient-centered and outcome-oriented approach in healthcare.

The healthcare industry in various regions must reconsider how the mentioned trends will impact their operational model and how they will most effectively engage in the M-Clinic 2.0 ecosystem. There is no one-size-fits-all solution for the healthcare industry, and each region will need to tailor its approach to best meet the needs of its patients. Therefore, M-Clinic 2.0 holds the potential to provide an excellent opportunity for the healthcare industry to improve the way it delivers care to patients.

The "Vaccine on Wheels" model was deployed during the peak of the third wave of COVID-19 between December 21 to March 22 by Jivika Healthcare. The model resulted in successful vaccination uptake by achieving the scale quickly and unlocking the demands by bringing socio-behavioral changes in the communities. The model has been revealed to be successful with the collaboration of government and various strategic/CSR partners in challenging and underserved regions. Based on the appreciation received from funders, administrations, state governments, development partners, and other prominent stakeholders, this model was recommended to be replicable in routine immunizations and other primary healthcare services. The strategic recommendations distinguished by longitudinal term impacts are summarized in Table 4. The recommendations are based upon the success factors of the VOW operational model, which led to a remarkable transformation in immunization coverage and other sustainable initiatives that can be implemented in the present structure of MMUs.

**Table 4:** Matrix of strategic recommendations.

Segments →	Tier-I	Tier-II	Rural/Tribal
Term ↓			
**Short-term**	• Collaborate with local hospitals and healthcare facilities to expand the services and provide a more comprehensive range of healthcare services. • Develop partnerships with local businesses and organizations to help support the operations and expand their reach. • Increase visibility in local communities by participating in health fairs and other public outreach events. • Consider expanding the hours of operation better to meet the needs of the patients in urban regions. • Explore ways to accept alternative forms of payment, such as insurance, to make the services more accessible to patients in urban regions.	• Partner with local NGOs and other organizations working in rural and semi-urban areas to help them with outreach and awareness campaigns about the various services offered by the mobile clinic. • Collaborate with local government bodies and panchayats to help set up and run these clinics smoothly with the infrastructure and logistical support required . • To increase the reach and impact, consider providing other allied health services, such as dental, optometry, etc., along with primary healthcare. • Collaborate with local pharmacies and diagnostic laboratories to provide discounts. • Use technology and social media platforms to create awareness about the services and reach out to a larger audience.	• Establish partnerships with local health facilities and providers to expand the range of services offered by MMUs. • Please work with the government to develop a sustainable funding model for MMUs to continue operating in rural and tribal regions. • Raise awareness about the services offered by MMUs among the target population so that more people can benefit. • Train healthcare providers working in MMUs in a broader range of primary healthcare services to offer patients more comprehensive care. • Implement quality assurance mechanisms to ensure that MMUs provide high-quality healthcare services to their communities.
**Medium-term**	• Establish partnerships with local hospitals and clinics to offer a broader range of services. • Expand the number of MMUs to cover more areas. • Offer more outreach programs to engage with the community and raise awareness about health services offered by MMUs. • Increase the number of trained staff and volunteers to offer more services. • Introduce new services such as mental health services, immunization programs, and health check-ups.	• Develop a business plan and financial model for expanding mobile clinic functions and services. • Develop partnerships with local health providers, NGOs, and other stakeholders to support expanding mobile clinic services. • Create a marketing and outreach plan to raise awareness of the mobile clinic and its services among the target population. • Train staff on the expanded functions and services the mobile clinic offers. • Implement a quality management system to ensure the provision of high-quality care.	• Establish permanent MMUs in rural and tribal regions of India. • Work with the government to expand the scope of services offered by MMUs. • Increase the number of MMUs operating in rural and tribal regions. • Educate communities about the advanced services offered by MMUs • Evaluate the effectiveness of MMUs in providing healthcare services. • Advocate for the expansion of MMUs in other rural and tribal regions.
**Long-term**	• Develop partnerships with local hospitals and clinics to refer patients for specialized care. • Offer a broader range of services such as dental care, mental health, and substance abuse counseling. • Increase outreach efforts to promote awareness of mobile clinic services in the community. • Develop a marketing strategy to target specific population groups in need of healthcare services. • Work with local government officials to identify areas in need of mobile clinic services. • Secure funding from donors and grant-making organizations to support expanded mobile clinic services.	• Create a comprehensive marketing and outreach strategy to raise awareness about the mobile clinic and its services that include more preventive and primary care services. • Increase the number of mobile clinic units to reach more patients in rural and underserved areas. • Increase the number of trained medical staff, including doctors, nurses, and community health workers, to provide care at mobile clinic units. • Invest in technology and infrastructure to improve the quality and efficiency of care delivered at mobile clinic units. • Develop and implement an evaluation framework to assess mobile clinic services' impact on patients' health outcomes in rural and underserved areas.	• Establish a clear and concise mission and goals for the mobile clinic program. • Conduct a needs assessment of the target population to identify the healthcare needs not being met by the current healthcare infrastructure. • Develop a business plan for the mobile clinic program that outlines the costs and revenue streams associated with providing expanded healthcare services. • Work with local and tribal leaders to develop relationships and secure buy-in for the mobile clinic program. • Train mobile clinic staff on delivering expanded healthcare services and develop protocols for providing these services. • Evaluate the mobile clinic program regularly to ensure that it meets the target population's needs and achieves the desired outcomes.

## 5 Conclusion

This study has aimed to understand and document the role of MMUs as an innovative intervention in enhancing healthcare accessibility, particularly during the COVID-19 pandemic. The qualitative analysis of in-depth interviews with healthcare professionals across various Indian states has emphasized the scalability, affordability, replicability, and sustainability of MMUs as critical components in their operational model. These dimensions have been recognized as pivotal in their effectiveness, especially in Tier I, II, and III regions, which face distinct healthcare challenges.

The findings emphasize the potential of MMUs as a cost-effective and adaptable solution for primary healthcare delivery in diverse settings, including resource-limited areas. The success of the VaccineOnWheels (VOW) program, particularly in rural and tribal communities, underscores the model's efficacy in reaching marginalized and hard-to-reach populations, thereby addressing healthcare disparities.

This research not only contributes to the academic understanding of service innovation in healthcare but also sets a foundation for future empirical studies. The identified factors - scalability, affordability, replicability, and sustainability - offer a broad framework for quantitative analysis and the development of logical models. Such empirical studies could further elucidate the dynamics and interrelationships of these components, enhancing the general concept of innovations in immunization and healthcare programs.

Moreover, the study provides valuable insights for policymakers and healthcare providers. It underscores the necessity of adopting innovative, equitable, and sustainable models like MMUs to transform healthcare systems globally. This approach is particularly pertinent for future primary healthcare initiatives in rural and tribal areas, where traditional healthcare services are often inadequate.

In conclusion, this study serves as a vital step in documenting the innovations in COVID-19 vaccination programs and sets the stage for further research and policy development in healthcare service innovation. It offers a robust platform for expanding our understanding and application of mobile healthcare solutions in various settings, contributing to the overarching goal of achieving equitable and comprehensive healthcare access for all.

## Funding

This study has been funded by the Bill and Melinda Gates Foundation.

## Ethics approval and consent to participate

Not applicable.

## Declaration of competing interest

The authors declare that they have no known competing financial interests or personal relationships that could have appeared to influence the work reported in this paper.

## References

[1] Wallerstein N., Duran B., Oetzel J.G., Minkler M. (2017). Community-based Participatory Research for Health: Advancing Social and Health Equity.

[2] Brown A., Edelman A., Pain T., Larkins S., Harvey G. (2022). “We*’*re not providing the best care if we are not on the cutting edge of research*”*: a research impact evaluation at a regional Australian hospital and health service. Int J Health Pol Manag.

[3] Ekezie W., Awwad S., Krauchenberg A. (2022). Access to vaccination among disadvantaged, isolated and difficult-to-reach communities in the WHO European region: a systematic review. Vaccines.

[4] Vashi A.P., Coiado O.C. (2021). The future of COVID-19: a vaccine review. Journal of infection and public health.

[5] Mishra V., Seyedzenouzi G., Almohtadi A. (2021). Health inequalities during COVID-19 and their effects on morbidity and mortality. J Healthc Leader.

[6] Núñez A., Sreeganga S.D., Ramaprasad A. (2021). Access to healthcare during COVID-19. Int J Environ Res Publ Health.

[7] Chanda S., Dogra V., Randhawa S. (2019). The design, operations, and feasibility of primary healthcare service delivery through mobile medical units. International Journal of Health Systems and Implementation Research.

[8] Khanna A.B., Narula S.A. (2017). Mobile medical units—can they improve the quality of health services in developing countries?. J Health Manag.

[9] Gebremeskel A.T., Otu A., Abimbola S., Yaya S. (2021). Building resilient health systems in Africa beyond the COVID-19 pandemic response. BMJ Glob Health.

[10] Gao M.Z., Chou Y.H., Chang Y.Z. (2022). Designing mobile epidemic prevention medical stations for the COVID-19 pandemic and international medical aid. Int J Environ Res Publ Health.

[11] Ho C.J., Khalid H., Skead K., Wong J. (2022). The politics of universal health coverage. Lancet.

[12] Gizaw Z., Astale T., Kassie G.M. (2022). What improves access to primary healthcare services in rural communities? A systematic review. BMC Primary Care.

[13] Bertoncello C., Cocchio S., Fonzo M., Bennici S.E., Russo F., Putoto G. (2020). The potential of mobile health clinics in chronic disease prevention and health promotion in universal healthcare systems. An on-field experiment. Int J Equity Health.

[14] Yu S.W., Hill C., Ricks M.L., Bennet J., Oriol N.E. (2017). The scope and impact of mobile health clinics in the United States: a literature review. Int J Equity Health.

[15] Tulledge‐Scheitel S., Bell S.J., Larsen J.J. (2021). A mobile unit overcomes the challenges to monoclonal antibody infusion for COVID‐19 in skilled care facilities. J Am Geriatr Soc.

[16] Rashmi M., Lekshmi V.N. (2021). Community mobilization during epidemic emergencies: insights from Kerala. Qual Soc Work.

[17] Sengupta P., Ganguli B., SenRoy S., Chatterjee A. (2021). An analysis of COVID-19 clusters in India: two case studies on Nizamuddin and Dharavi. BMC Publ Health.

[18] Hall M.A., Wright R.F. (2008). Systematic content analysis of judicial opinions. Calif Law Rev.

[19] Raut A., Samad A., Verma J., Kshirsagar P. (2023). Acceptance, hesitancy and refusal towards COVID-19 vaccination. Clinical Epidemiology and Global Health.

[20] Dhankher R., Mukhopadhyay A., Bhowmick S. (2023). Perception regarding COVID-19 vaccine and COVID appropriate behavior among adolescents at a tertiary hospital, West Bengal: a longitudinal survey. Clinical Epidemiology and Global Health.

[21] Goel K., Sen A., Goel P. (2022). Community health workers willingness to participate in COVID-19 vaccine trials and intention to vaccinate: a cross-sectional survey in India. Clinical Epidemiology and Global Health.

[22] Panda S., Dash M., Mishra R. (2023). Voice from the frontline and learning for the future: a qualitative descriptive study on wider perspectives of frontline nurses in India during the COVID 19 global pandemic. Clinical Epidemiology and Global Health.

